# hNGF Peptides Elicit the NGF-TrkA Signalling Pathway in Cholinergic Neurons and Retain Full Neurotrophic Activity in the DRG Assay

**DOI:** 10.3390/biom10020216

**Published:** 2020-02-01

**Authors:** Viviana Triaca, Elena Fico, Valentina Sposato, Silvia Caioli, Maria Teresa Ciotti, Cristina Zona, Delio Mercanti, Diego La Mendola, Cristina Satriano, Enrico Rizzarelli, Paola Tirassa, Pietro Calissano

**Affiliations:** 1 Institute of Biochemistry and Cell Biology, National Research Council (CNR-IBBC), International Campus A. Buzzati Traverso, Via E. Ramarini 32, Monterotondo, 00015 Rome, Italy; 2 Institute of Biochemistry and Cell Biology, National Research Council (CNR-IBBC), at Department of Sense Organs, University of Rome “ La Sapienza”, Viale del Policlinico 155, 00161 Rome, Italy; ficoelena275@gmail.com (E.F.); teresa.ciotti@ibcn.cnr.it (M.T.C.); delio.mercanti@cnr.it (D.M.); paola.tirassa@cnr.it (P.T.); 3 European Brain Research Institute (EBRI Foundation), Viale Regina Elena 295, 00161 Rome, Italy; v.sposato@ebri.it (V.S.); p.calissano@ebri.it (P.C.); 4 IRCCS S. Lucia Foundation, Via del Fosso di Fiorano 64, 00143 Rome, Italy; silviacaioli@yahoo.it (S.C.); zona@uniroma2.it (C.Z.); 5 Department of Systems Medicine, University of Rome “TorVergata”, Via Montpellier 1, 00133 Rome, Italy; 6 Department of Pharmacy, University of Pisa, via Bonanno Pisano 6, 56126 Pisa, Italy; lamendola@farm.unipi.it; 7 Department of Chemical Sciences, University of Catania, Viale Andrea Doria 6, 95125 Catania, Italy; csatrian@unict.it (C.S.); erizzarelli@unict.it (E.R.); 8 Institute of Crystallography, National Research Council (CNR-IC), Via Paolo Gaifami 18, 95126 Catania, Italy

**Keywords:** NGF mimetic, hNGF1–14, TrkA agonist, cholinergic neurons, DRG, neurotrophic therapy

## Abstract

In the last decade, Nerve Growth Factor (NGF)-based clinical approaches have lacked specific and efficient Tyrosine Kinase A (TrkA) agonists for brain delivery. Nowadays, the characterization of novel small peptidomimetic is taking centre stage in preclinical studies, in order to overcome the main size-related limitation in brain delivery of NGF holoprotein for Central Nervous System (CNS) pathologies. Here we investigated the NGF mimetic properties of the human NGF 1–14 sequence (hNGF1–14) and its derivatives, by resorting to primary cholinergic and dorsal root ganglia (DRG) neurons. Briefly, we observed that: 1) hNGF1–14 peptides engage the NGF pathway through TrkA phosphorylation at tyrosine 490 (Y490), and activation of ShcC/PI3K and Plc-γ/MAPK signalling, promoting AKT-dependent survival and CREB-driven neuronal activity, as seen by levels of the immediate early gene c-Fos, of the cholinergic marker Choline Acetyltransferase (ChAT), and of Brain Derived Neurotrophic Factor (BDNF); 2) their NGF mimetic activity is lost upon selective TrkA inhibition by means of GW441756; 3) hNGF1–14 peptides are able to sustain DRG survival and differentiation in absence of NGF. Furthermore, the acetylated derivative Ac-hNGF1–14 demonstrated an optimal NGF mimetic activity in both neuronal paradigms and an electrophysiological profile similar to NGF in cholinergic neurons. Cumulatively, the findings here reported pinpoint the hNGF1–14 peptide, and in particular its acetylated derivative, as novel, specific and low molecular weight TrkA specific agonists in both CNS and PNS primary neurons.

## 1. Introduction

Nerve Growth Factor (NGF) is the prototypic neurotrophin primarily described for its ability to sustain survival and induce differentiation of peripheral neurons [1]. Over the last decades, NGF has been demonstrated to act also on the Central Nervous System (CNS), exerting pleiotropic actions beyond the nervous system, including the immune and the endocrine systems [2].

NGF homodimers bind to a multireceptor complex composed of two main classes of receptors, namely the tropomyosin receptor kinase A (TrkA) and the pan neurotrophin (p75^NTR^) receptor, to regulate the growth, development, survival and repair of the nervous system. TrkA engagement by its ligand induces receptor dimerization and autophosphorylation at the tyrosine residue 490 (Y490), driving the activation of early and downstream signalling molecules, such as 1) phospholipase C-γ (PLC-γ) pathways leading to intracellular calcium release, ion channels and transcription factors expression, 2) neuronal SH2 containing protein (ShcC)-driven phosphatidylinositol 3-kinase (PI3k) activation, which is critical to inhibit c-Jun N-terminal kinase (JNK) induced apoptosis by activating AKT-mediated survival, and 3) mitogen-activated protein kinase (MAPK)/Extracellular Signal-regulated Kinase (ERK) route. MAPK/ERK promote neuronal differentiation and survival [3,4] by cAMP response element binding protein (CREB) mediated gene transcription, resulting in stimuli-dependent expression of the early immediate gene c-Fos [5]. The p75 receptor activates pro-survival pathways, such as AKT, to act synergistically with Trk-mediated neurotrophin effects. Alternatively, it can operate in an antagonistic manner to cell survival and growth, triggering several potential pro-apoptotic cascades [1,2].

NGF has been implicated in the therapy of a broad range of age-related, neuroinflammatory or genetic neuropathologies, like Alzheimer’s Disease, Parkinson’s Disease, autism, glaucoma, multiple sclerosis, Down’s syndrome, spinal cord injury, traumatic brain injury, and type 2 diabetes [2,6,7,8,9,10]. Despite the therapeutic potential of NGF, clinical trials featuring this protein have been disappointing. Consequently, the design of small stable peptides with NGF-specific effects, better pharmacokinetics, and fewer side effects is sought nowadays. Unfortunately, most of the available drugs are aspecific inducers of neurotrophic pathways or only partial TrkA agonists. Thus, the design of novel targeted, effective and specific small NGF-like compounds is of major relevance for the advancement of therapeutic approaches to neurodegenerative diseases.

Accordingly, several attempts have been made to recapitulate NGF properties by means of short NGF native peptides as well as by designing synthetic products [11,12,13]. Peptide mapping, mutagenesis, crystallography and molecular modelling studies helped in the identification of the N-terminus residues 1–8 and hydrophilic β-hairpin loops 1,2 and 4 as candidate TrkA binding sites [14,15,16,17,18,19,20,21,22,23,24,25]. As for the NGF binding sites in the TrkA receptor, three key recognition sites modulate ligand binding and receptor activation. As shown by crystallization studies, the human TrkA domain 5 (TrkA-D5) within the IgC2 region is the main TrkA activation site, critical for NGF binding and biological activity [26]. The N-terminal domain of NGF binds to TrkA-D5 due to several non-covalent aromatic interactions strengthening their binding through histidines 4 and 8 (His4, His8) [27,28,29,30]. Each neurotrophin N-terminus is known to stabilize the respective Trk-D5 peptide, regulating both the affinity and specificity of neurotrophin-receptors pairs, and accounts for limited cross-reactivity among neurotrophin signalling systems [29,30,31]. In line with this notion, no significant binding of BDNF to the TrkA-D5 domain has been reported [32,33], while NGF and NT3 bind to their respective receptors TrkA and TrkC receptors through distinct sites within the D5 domain [21,34]. Interestingly enough, TrkA-D5 has been proved to be a good therapeutic agent in animal models of asthma and pain [35] and can be envisaged as a druggable target in neuroprotective therapies.

The small N-terminal 1–14 sequence of the human NGF (hNGF1–14) has been previously shown to bind TrkA-D5 similarly to NGF through several non-covalent interactions [28,36,37]. Recently, hNGF1–14 has been prospected to mimic the whole NGF protein by activating TrkA and its downstream pathway in PC12 and SH-SY5Y neuroblastoma cell lines, mainly in presence of Cu^2+^ and Zn^2+^ metals [27,36].

We found that hNGF1–14 retains biological activity of the NGF protein, activating the early and late signalling intermediates of the NGF-TrkA pathway, and finally results in CREB-driven gene transcription and Immediate Early Gene c-Fos nuclear elevation. Among downstream effects, hNGF1–14 sustains ChAT level, excitatory neurotransmitter release, and promotes mature BDNF expression in cholinergic cultures. As a confirmation of its NGF-like activity, hNGF1–14 is also able to promote survival and differentiation of primary dorsal root ganglia (DRG) neurons in the classical bioassay. Overall, present and previous data envisage the in vivo preclinical studies with hNGF1–14 peptides as potent and selective NGF mimetics, and TrkA agonists for CNS and PNS in age-related or inflammation-driven human neurodegenerative diseases.

## 2. Materials and Methods

### 2.1. hNGF Peptides Synthesis

The human NGF sequence SSSH^4^PIFH^8^RGESFV-NH_2_ (monomeric hNGF1–14 peptide), the acetylated monomer Ac-SSS H^4^PIFH^8^RGESFV-NH_2_ (Ac-hNGF1–14 peptide), the (SSS H^4^PIFH^8^RGESFC-S)_2_ hNGF1–15 dimer, and the hNGF1–14 scramble peptide (GFRESPHVSISSH-NH2) with the amidated C-terminal were synthesized at the University of Catania, Italy. Of note, the hNGF1–14 peptides contain two histidine residues (His4 and His8) implicated in TrkA binding and NGF biological activity [25]. These two conserved histidines are misplaced in the scrambled peptide.

Peptides synthesis, purification, and spectroscopic characterization and docking simulation have been previously described [29,38,39]. Briefly, the peptides were synthesized by solid phase peptide synthesis (SPPS) on a Pioneer Peptide Synthesiser^TM^ (Applied Biosystems Waltham, MA, USA). The amino acid residues were coupled each other and to the TGR resin (Novabiochem®, Merck KGaA, Darmstadt, Germany, substitution 0.25 mmol/g, 0.1 mmol scale synthesis, 0.4 g of resin), by using 2-(1*H*-benzotriazole-1-yl)-1,1,3,3-tetramethylaminium tetra-fluoroborate (TBTU) (4 equivalents/amino acid), hydroxybenzotriazole (HOBT) (4 equivalents/amino acid), *N*,*N*-diisopropylethylamine (DIEA) (8 equivalentd/amino acid). All the reagents were solubilised in *N*,*N*-dimethylformamide (DMF). Removal of fluorenylmethyloxycarbonyl protecting group (Fmoc) during synthesis was achieved by means of 20% piperidine solution in DMF.

A monomeric derivative was obtained by N-terminal acetylation to remove the positive charge and prevent N-terminal degradation. N-terminal acetylation was performed by treating the fully assembled and protected peptide resins, after removal of the N-terminal Fmoc group, with a solution containing acetic anhydride (6% *v*/*v*) and DIEA (5% *v*/*v*) in DMF. The peptides were cleaved off from their respective resins and simultaneously deprotected by treatment with a mixture of trifluoroacetic acid (TFA)/triisopropylsilane (TIS)/H_2_O (95/2.5/2.5% *v*/*v*) for 2 h at room temperature (RT). Solution containing the resin-released peptide was filtered off and concentrated in vacuum at 30 °C. The peptide was precipitated with cold freshly-distilled diethyl ether. The precipitate was then filtered, dried under vacuum, re-dissolved in water and lyophilised.

### 2.2. hNGF Peptides and NGF Purification

Peptides were purified by means of reverse-phase high-performance liquid chromatography by using a PrepStar 200 model SD-1 chromatography system (Varian, Palo Alto, CA, USA). NGF protein was purified from rodent submaxillary glands, as already reported [38,39,40].

### 2.3. Reagents and Antibodies

Reagents: picrotoxin and strychnine were purchased from Sigma (St. Louis, MO, USA) and were dissolved in ethanol (96%, Sigma) and water, respectively. Tetrodotoxin (TTX; Alomone Labs, Jerusalem, Israel) was dissolved in water.

Primary Antibodies: anti-pTrkA^Y490^ (Cell Signalling, Danvers, MA, USA, 9141), anti-Trk (Santa Cruz, Santa Cruz, CA, USA, sc7268), anti-pShc^Y317^ (Cell Signalling, 2434), anti-ShcC (Cell Signalling, 2431), anti-pPLC-γ ^Y783^ (Cell Signalling, 2821), anti PLC-γ (Cell Signalling, 14008), anti-pMAPK^Y202/Y204^ (Cell Signalling, 9101), anti-MAPK (Cell Signalling, 9102), anti-pPI3K^Y458/Y199^ (Cell Signalling, 4228), anti-pAKT^S473^ (Cell Signalling, 4051), anti-AKT (11E7) (Cell Signalling, 4685), anti-pCREB^S133^ (Millipore, Temecula, CA, USA, 06-519), anti-BDNF (Millipore, MABN110), anti-c-Fos (Cell Signalling, Rb Mab 2250S), anti-ChAT (Millipore, AB144P), and anti-NeuN (ABCAM, city, state abbrev if USA, country ab177487), anti-MAP2 (Cell Signalling, 4542; Millipore, MAB3418). Secondary antibodies: anti mouse-HRP and anti rabbit-HRP (PerkinElmer, Waltham, MA, USA); Donkey anti mouse-Alexa546, donkey anti rabbit-Alexa488 (Life Technologies, Carlsbad, CA, USA).

### 2.4. Dorsal Root Ganglion Dissociated Culture

(DRG dissociated primary neurons were prepared from neonatal (2 days, P2) Wistar rats (Charles River, Wilmington, MA, USA) [41,42]. All animal procedures were approved by the Ethics Committee of the CERC. Animals were handled in compliance with the national (D.Lgs26/2014) and European Union legislation guidelines for animal welfare (2010/63/EU). All efforts were made to minimize the number of animals used and suffering. 

Briefly, DRG were collected, incubated for 1 h at 37 °C with 0.125% collagenase (Sigma-Aldrich), mechanically dissociated and plated onto coverslips or Petri dishes pretreated with 10 mg/mL poly-l-lysine (Sigma-Aldrich), at a density of 50,000 cells/well of a 24 well tissue culture plate. The DRG cultures were maintained in low-serum medium, consisting of Dulbecco’s modified Eagle’s medium (DMEM)/F12 (Invitrogen, Carlsbad, CA, USA) supplemented with 0.05% N_2_ (Invitrogen), 87.5 ng/mL 5-fluoro-20-deoxyuridine, 37.5 ng/mL uridine, 50 U/mL penicillin and 50 mg/mL streptomycin (Sigma-Aldrich) at 37 °C in 5% CO_2_. After 2 days in vitro, DRG cultures were stimulated with 10 μM scramble peptide, 10 μM hNGF1–14 peptide, 10 μM Ac-hNGF1–14 peptide, 10 μM hNGF1–15 dimer or NGF (100 ng/mL; 3.84 nM) for 5 days. Unstimulated DIV2 neurons maintained in basal medium conditions for 5 days were considered as control DRG neurons (CTR). After the treatments, neurons were quickly washed with PBS, PFA fixed (4%, 15′, RT) and kept in PBS at 4 °C for immunofluorescent staining.

### 2.5. Cholinergic Neurons Culture

Cholinergic neurons were harvested from the septal region of E17 Wistar rat embryos, as previously described [39,40] and dissociated cells were seeded as follows: 1.5 × 10^6^ cells on poly-l-lysine (Sigma) coated 35 mm plates (BD Falcon, Durham, NC, USA; 353001) for western blotting analyses and 5 × 10^4^ cells on glass coverslips in 24-wells plates (BD Falcon; 351147) for immunofluorescence analysis. Cells were grown in Neurobasal (NB) medium supplemented with 2% B27 (Invitrogen) in a 5% CO_2_ incubator at 37 °C (ThermoFisher Scientific, Waltham, MA, USA) for 10 days and then used for treatments (DIV10). Cholinergic neurons were treated with murine NGF (100 ng/mL; 3.84 nM, 30′), 10 μM scramble peptide, 10 μM hNGF1–14 peptide, 10 μM Ac-hNGF1–14 peptide or 10 μM hNGF1–15 dimer in NB medium supplemented with 2% B27. DIV 10 untreated cholinergic neurons were the control neurons (CTR) in this study. After the treatments, neurons were quickly washed with PBS and kept at −20 °C until biochemical analysis or PFA fixed (4%, 15′, RT) and kept in PBS at 4 °C for immunofluorescent staining.

### 2.6. Western Blotting

Cultured neurons were digested in a RIPA buffer with “complete protease and phosphatase inhibitory cocktail” (Roche, Basel, Switzerland) and centrifuged (10,000 rpm, 20′). The supernatants were collected, and the amount of total protein was determined by Quick Start Bradford Dye Reagent. Each sample (40 μg) was separated by SDS-PAGE in precast 4–12% Bis-Tris Plus gels (Bolt, Invitrogen), transferred to nitrocellulose membranes (0.45 μM, GE Healthcare, Chicago, IL, USA), and incubated for 1 h at RT with 10% non-fat dry milk in TBS-T (10 mM Tris, pH 7.5, 100 mM NaCl, and 0.1% Tween-20). The overnight incubation with primary antibody (4 °C) was followed by incubation with the appropriate HRP-conjugated secondary antibody (1:2000, 1 h, RT, ThermoFisher Scientific, Waltham, MA, USA) and the ECL substrate (32106; ThermoFisher Scientific, Waltham, MA, USA). The films were digitalized using a professional scanner (HP4050) and quantified by gel densitometry using the ImageJ software (NIH, Bethesda, MD, USA). Measurements were standardized between the experimental groups using the same calibration system and a fixed threshold over the background. To measure the effect of NGF/peptides on the activation of the pathway, the phosphoprotein level was normalized over the total protein level, with a further normalization over β-actin to correct for sample loading bias [43]. Data are expressed as percentage optical density relative to control group, and presented as mean ± SEM.

### 2.7. Immunofluorescence Labelling and Microscopy

Primary cultures were fixed for 20′ in PBS containing 4% paraformaldehyde, permeabilised with PBS plus 0.3% Tween, and quenched by ammonium chloride (50 mM, 30′, RT). Aspecific staining by the secondary antibody was blocked by incubation with normal donkey serum (10%, 1 h, RT). The overnight incubation (4 °C) with primary antibodies was followed by the appropriate fluorescent secondary antibodies (1:2000, 1 h, RT). 

For pAKT/pCREB double immunofluorescence, a mixture of rabbit anti-pAKT (1:500) and mouse anti-pCREB (1:100) antibodies was followed by incubation with a mixture of donkey anti-rabbit Alexa488 and anti-mouse Alexa546 secondary antibodies. The single c-Fos and pCREB immunostainings of cholinergic neurons were performed with rabbit anti-c-Fos (1:500) or rabbit anti-pCREB (1:200) antibodies, followed by anti-rabbit AlexaFluor-488 antibody (1:2000, 1 h, RT). MAP2 staining of DRG neurons was achieved by means of mouse anti-MAP2 antibody (Cell Signalling, 1:100, ON, 4 °C) followed by anti-mouse Alexa546 antibody (1:2000, 1 h, RT). The double pCREB/MAP2 immunostaining of cholinergic neurons were performed by a mixture of rabbit anti-pCREB (1:100) and mouse anti-MAP2 antibodies (Millipore, 1:500, ON, 4 °C), followed by incubation with donkey anti-rabbit Alexa488 and donkey anti-mouse Alexa546 antibodies.

Nuclei were counterstained with 4′,6-diamidino-2-phenylindole (DAPI; 1:1000; 15 min, RT; Life Technologies). Then, coverslips were mounted on Superfrost glass slides using the Prolong Gold Antifade Mounting (Life Technologies) and kept at −20 °C before image analysis.

Immunofluorescence images were acquired with an epifluorescent microscope (Leica CTR5500; Leica Microsystems, Mannheim, Germany) equipped with a CCD camera (Leica) or by confocal microscopy with the laser scanning confocal microscope TCS SP5 (Leica Microsystems) using a 40× (NA = 1.25), and a 63× (NA = 1.4) oil-immersion lens. A UV Diode laser operating at 405 nm, an argon laser at 488 nm, a HeNe laser at 543 nm were used as excitation sources. 

NeuN widefield mosaic images were acquired with an inverted microscope (DMI6000 Leica Microsystems) equipped with a sCMOS camera (Zyla, Andor, Oxford Instruments, Oxon, UK) and a white light lamp. Image acquisition was controlled with Leica Application Suite (LAS X software, version 3.7.0.20979, Leica Microsystems, Mannheim, Germany). Mosaics were generated by merging several individual frames, using an automated spatial overlap of 15% with LAS X software (Leica MicroSystems, Mannheim, Germany). 

### 2.8. Neuronal Counts

Immunofluorescent images were collected for direct comparison among experimental groups using fixed settings. The number of positive cells over the total number of DAPI stained nuclei per field (field area = 0.366 μm^2^; 20× objective) was measured. The analysis was performed after calibrating for particle pixels size (50-400 pixels) and applying a fixed threshold over the background. Nuclei were counted both by manual and automated counting methods (ImageJ) with comparable results. Data are expressed as percentage to control group (CTR) and presented as mean ± SEM.

### 2.9. Analysis of DRG Bioassay

DRG neurons were fixed and permeabilized in 4% paraformaldehyde with 0.2% Triton X-100 in 0.1M Tris-HCl (pH = 7.4) for 5′, and immunolabelled for MAP2, as described above. Neurons with neurites connected to the soma, longer than the mean neuronal soma diameter, typically around 25 μM, and with an identifiable axonal arborisation were quantified for a number of parameters. In detail, the number of neurons, total number of neurites, total neurite length (sum of lengths of all neuritis in the field), maximum neurite length (length of longest neurite), and total number of branch points were measured and compared by automated cell count by means of ImageJ.

A threshold over the background was fixed by applying a contrast-based mask, followed by the calibration for particle pixels size (50–400 pixels) to exclude aspecific pins. Number of cell bodies was automatically counted by Image J, while neurites were drawn and quantified by means of the NeuronJ plugin (ImageJ). 

### 2.10. Electrophysiology

Whole cell patch-clamp recordings were performed from primary neurons in cholinergic cultures (E17, DIV10) to study the excitatory neurotransmission in different culture conditions. The recording pipettes were pulled from borosilicate glass with an outer diameter of 1.2 mm and had open tip resistances of 3–5 MΩ. The internal solution for filling pipettes consisted of 140 mM CsCl, 1 mM EGTA, 10 mM HEPES and 6 mM d-glucose (pH 7.4 with CsOH). The standard extracellular solution consisted of 130 mM NaCl, 3 mM KCl, 2 mM MgCl_2_, 1.5 mM CaCl_2_, 10 mM HEPES, 6 mM d-glucose, and 10 mM tetraethylammonium (TEA) Cl (pH 7.4 with NaOH). 

To isolate the miniature Excitatory Post Synaptic Currents (mEPSCs), 0.5 μM tetrodotoxin (TTX), 5 μM strychnine, 100 μM picrotoxin were added in the bath solution, in order to block voltage-dependent Na+ currents, glycine and GABAA receptors, respectively. Recordings were carried out for 5′ from each neuron and the last 2′ of each recording were analysed.

In order to evaluate the effect of Ac-hNGF1–14 peptide on mEPSCs, Ac-hNGF1–14 peptide and NGF were added to the culture medium for 30′ and maintained at 37 °C. Specifically, recordings were obtained from neurons in control condition (Control), following treatment with the Ac-hNGF1–14 peptide (Ac-hNGF1–14; 10 µM, 30′) and following treatment with NGF (NGF; 100 ng/mL; 3.84 nM, 30′). As negative control respectively for TrkA requirement and sequence specificity, neurons were incubated the TrkA specific inhibitor GW441756 (15 μM, 45′) followed by NGF treatment (NGF; 100 ng/mL; 3.84 nM, 30′), or with the scrambled hNGF1–14 (Scr; 10 μM, 30′). The Ac-hNGF1–14 peptide, the scrambled hNGF1–14 and NGF were also added to the extracellular solution, according to the experimental condition.

Experiments were performed at 22–24 °C, RT. Recordings were made using a MultiClamp 700B amplifier (Molecular Devices, San Jose, CA, USA). pCLAMP 9.2 software was utilized for the data acquisition system (Molecular Devices, San Jose, CA, USA). After the formation of a high-resistance seal (>1 GΩ), the capacitance and the resistance of the electrodes were compensated electronically. The whole cell capacitance was assessed online using the Membrane test function of pClamp9.2. Current signals were sampled at 100 kHz and filtered at 3 kHz. 

### 2.11. Viability Assay

Neuronal viability was assessed by the 3-(4,5-dimethylthiazol-2-yl)-2,5-diphenyltetrazolium bromide (MTT) assay, according to manufacturer’s instructions (Invitrogen/Molecular Probes, Eugene, OR, USA), and with Trypan blue exclusion assay, as previously described [40], with comparable results.

The MTT assay is based on reduction of MTT to an insoluble formazan product by viable and metabolically active cells. DIV10 neurons, previously seeded on 96-wells microplates, were treated for 1 h with hNGF1–14 peptides at different concentrations (2, 5, 10, 50, 100 μM), or untreated (CTR) DIV10 neurons were incubated with MTT (0.5 mg/mL, 20′, 37 °C) in Hank’s balanced salt solution (Life Technologies). The formazan crystals were dissolved in dimethyl sulphoxide (DMSO), and the absorbance at 570 nm wavelength was measured using a Bio-Rad microplate reader (Bio-Rad, Hercules, CA, USA). The amount of MTT conversion was evaluated as a percentage of the absorbance measured in treated cells relative to the absorbance of control cells.

Alternatively, the Trypan Blue test allows the count of living cells able of cytoplasmic dye exclusion. Briefly, DIV10 neuron seeded on glass coverslips in 24-wells plates and treated for 1 h with hNGF1–14 peptides at different concentrations (2, 5, 10, 50, 100 μM), or untreated (CTR) DIV10 neurons were incubated with Trypan Blue (4%, 15′, RT), and then cleared, and mounted on glass slides. The number of Trypan blue positive dying cells was counted, converted into percentage of surviving cells and reported in the graphs (Supplementary Figure S1).

### 2.12. Statistical Analysis

The graphs were generated using PRISM (GraphPad Software, Inc., San Diego, CA, USA), and the data reported as the mean ± standard error of the mean (SEM). One-way ANOVA followed by Student’s t-test or Tukey-Kramer post-hoc was used to analyze the data, depending on the number of variables and groups (Statview-SAS, Cary, NC, USA). A *p* value <0.05 was considered statistically significant. 

To analyse miniature Excitatory Post Synaptic Currents (mEPSCs), the 6.0.7 version of Mini Analysis Program (Synaptosoft Inc., Decatur, GA, USA) was used. mEPSCs were manually detected using a 8 pA threshold crossing algorithm. Frequency, event amplitude, kinetic characteristics (rise and decay time), and event area were compared in the different experimental conditions. Data were expressed as mean ± SEM. Fitting and statistical analysis were performed using SPSS 17.0.0 for Windows (SPSS Inc., Chicago, IL, USA) and Origin 7.0 (Microcal Software, Northampton, MA, USA). Statistical tests were performed by using One-Way ANOVA followed by Bonferroni post-hoc correction. Statistical significance was taken as *p* < 0.05.

## 3. Results

### 3.1. hNGF1–14 Peptides Activate both TrkA-Shc and TrkA-PLC-γ Signalling Pathways in Cholinergic Neurons

In order to address hNGF1–14 peptides as NGF signalling agonists, we resorted to a well-established and characterized in vitro method of cholinergic neurons culture [41,42,44]. We found that the hNGF1–14 peptides exhibited NGF-like properties at micromolar concentrations, activating TrkA-related signal transduction and mimicking NGF neurotrophic effects, whereas the scrambled sequence peptide showed any NGF mimetic activity and was comparable to control, and confirmed hNGF1–14 sequence specificity. 

**Figure 1:**
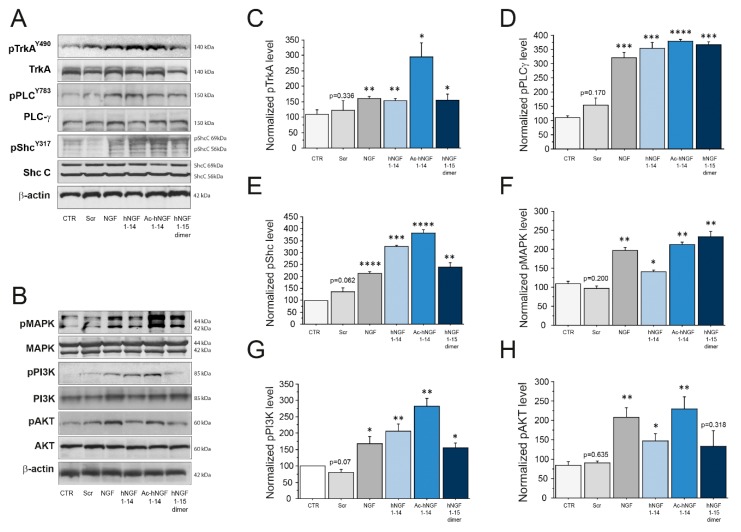
Activation of the NGF-TrkA signalling pathway in cholinergic neurons by hNGF1–14 peptides. (**A**, **C**–**E**) Representative western blotting (**A**) and densitometric analyses (**C**–**E**) of pTrkA (**A**,**C**), and early Trk signalling adaptors pPLC-γ (**A**,**D**), pShc (**A**,**E**) in cholinergic neurons treated for 7′ with 10 μM scrambled hNGF1–14 (Scr), 10 μM hNGF1–14, 10 μM Ac-hNGF1–14, 10 μM hNGF1-15 dimer and 100 ng/mL (3.84 nM) NGF. (**B**, **F**–**H**). Representative western blotting (**B**) and densitometric analyses (**F**–**H**) of pMAPK (**B**,**F**), pPI3K (**B**,**G**), pAKT (**B**,**H**) in cholinergic neurons treated for 15-20′ with 10 μM scrambled hNGF1–14 (Scr), 10 μM hNGF1–14, 10 μM Ac-hNGF1–14, 10 μM hNGF1–15 dimer and 100 ng/mL (3.84 nM) NGF. The phosphorylated level of each signalling molecule was reported as ratio over the corresponding total protein, and further normalized using β-actin as loading control. Data from *n* = 3 independent experiments were expressed as percentage of CTR and reported as mean +SEM. * *p* < 0.05; ** *p* < 0.01; *** *p* < 0.001; **** *p* < 0.0001. Full-length blots are presented in the Additional file.

In detail, cholinergic neurons were incubated with 10 μM scrambled hNGF1–14, 10 μM hNGF1–14, 10 μM Ac-hNGF1–14, and 10 μM hNGF1–15 dimer either for 7′ to study activation of the NGF specific neurotrophic receptor TrkA (Figure 1A,C) and early adaptors PLC-γ (Figure 1A,D) and ShcC (Figure 1A,E), or for 15–20′ to analyse downstream effectors, like MAPK (Figure 1B,F), PI3K (Figure 1B,G) and AKT (Figure 1B,H). Murine NGF (NGF; 100 ng/mL, equivalent to 3.84 nM) was used as positive control.

The analysis of TrkA activation (Figure 1A,C) showed that NGF significantly increased pTrkA level (NGF; 161.31 ± 7.24% of CTR; ** *p* < 0.01), as well as hNGF1–14 (153.03 ± 7.59% of CTR; ** *p* < 0.01), Ac-hNGF1–14 (296.21 ± 44.49% of CTR; * *p* < 0.05), and hNGF1–15 dimer (155.36 ± 18.80% of CTR; * *p* < 0.05). No statistically significant difference was found between the control (87.67 ± 9.50%) and the scrambled hNGF1–14 (122.48 ± 30.45% of CTR; *p* = 0.336) neurons.

The level of PLC-γ (Figure 1A,D) was stimulated by NGF (NGF; 321.08 ± 18.66% of CTR; *** *p* < 0.001), as well as with hNGF1–14 (353.60 ± 21.98% of CTR; *** *p* < 0.001), Ac-hNGF1–14 (379.10 ± 6.90% of CTR; **** *p* < 0.0001), and hNGF1–15 dimer (366.36 ± 10.43% of CTR; *** *p* < 0.001). No statistically significant difference was found between control (110.33 ± 6.06%) and scrambled hNGF1–14 (154.40 ± 25.64% of CTR; *p* = 0.170) neurons.

Also, the activation of pShc (Figure 1A,E) was upregulated upon treatment with NGF (NGF; 214.24 ± 4.94% of CTR; **** *p* < 0.0001), as well as with hNGF1–14 (326.85 ± 4.43% of CTR; *** *p* < 0.001), Ac-hNGF1–14 (381.58 ± 15.33% of CTR; **** *p*< 0.0001), and hNGF1–15 dimer (239.95 ± 17.01% of CTR; ** *p* < 0.01). No statistically significant difference was found between control (CTR: 98.00 ± 1.16) and scrambled hNGF1–14 (138.85 ± 15.83% of CTR; *p* = 0.062) treated neurons.

As for downstream signalling molecules, MAPK phosphorylation (Figure 1B,F) was augmented by NGF (NGF; 197.54 ± 6.38% of CTR; ** *p* < 0.01), as expected. Interestingly, also hNGF1–14 (141.15 ± 3.67% of CTR; * *p* < 0.05), Ac-hNGF1–14 (213.14 ± 5.09% of CTR; ** *p* < 0.01), and hNGF1–15 dimer (222.92 ± 14.02% of CTR; ** *p* <0.01 but not the scrambled hNGF1–14 (97.27 ± 5.27% of CTR; *p* = 0.200) were able to stimulate MAPK, as compared to control (CTR: 109.67 ± 6.12%).

Also pPI3K level (Figure 1B,G) increased following NGF (NGF; 166.88 ± 23.58% of CTR; * *p* < 0.05), as well as with hNGF1–14 (205.98 ± 21.65% of CTR; * *p* < 0.01), Ac-hNGF1–14 (282.46 ± 23.49% of CTR; ** *p* < 0.01), and hNGF1–15 dimer (155.60 ± 13.85% of CTR; * *p* < 0.05). No statistically significant difference was found between control (CTR: 100 ± 0%) and scrambled hNGF1–14 (80.12 ± 8.3% of CTR; *p* = 0.07) neurons.

The survival factor AKT (Figure 1B,H) was stimulated by NGF (NGF; 207.96 ± 24.83% of CTR; ** *p* < 0.01), as expected, as well as by the hNGF1–14 (147.19 ± 17.65% of CTR; * *p* < 0.05), and Ac-hNGF1–14 (230.45 ± 30.04% of CTR; ** *p* < 0.01). No statistically significant difference was seen upon treatment with the hNGF1–15 dimer (132.38 ± 40.82% of CTR; *p* = 0.318). The scrambled hNGF1–14 didn’t exert any effect on pAKT level (90.25 ± 5.78% of CTR; *p* = 0.635), by comparison with control neurons (84.64 ± 9.29%).

To assess cell viability following hNGF1–14 peptides incubation, number of surviving neurons was evaluated after escalating doses (2, 5, 10, 50, 100 μM; Supplementary Figure S1) of scrambled peptide (Supplementary Figure S1A; 2 μM: *p* = 0.606, 5 μM: *p* = 0.427, 10 μM: *p* = 0.934, 50 μM: *p* = 0.597, 100 μM: *p* = 0.869), hNGF1–14 (Supplementary Figure S1B; 2 μM: *p* = 0.959, 5 μM: *p* = 0.698, 10 μM: *p* = 0.549, 50 μM: *p* = 0.556, 100 μM: *p* = 0.601), Ac-hNGF1–14 (Supplementary Figure S1C; 2 μM: *p* = 0.449, 5 μM: *p* = 0.805, 10 μM: *p* = 0.655, 50 μM: *p* = 0.447, 100 μM: *p* = 0.758) and hNGF1–15 dimer (Supplementary Figure S1D, 2 μM: *p* = 0.917, 5 μM: *p* = 0.989, 10 μM: *p* = 0.815, 50 μM: *p* = 0.854, 100 μM: *p* = 0.514) peptides. At 10 μM concentration the peptides fully exploit a NGF-like action (Supplementary Figure S2A,B), inducing the TrkA pathway and downstream activation of CREB/c-Fos in cholinergic neurons (A), sustaining survival and neurites extension of dissociated DRG neurons, as seen by MAP2 immunolabelling (B). To address sequence specificity requirement for biological activity of the hNGF1–14 peptide, scrambled hNGF1–14 sequence was used as negative control (Supplementary Figure S1A).

Overall, these findings pinpoint that hNGF1–14 at 10 μM concentration exerts the maximum efficacy in terms of activation of the NGF receptor TrkA without affecting neuronal viability. For these reasons, 10 μM was the working concentration of the hNGF1–14 peptides in this study.

### 3.2. hNGF1–14 Peptides Administration Increases the Number of Cholinergic Neurons Expressing pAKT and Nuclear pCREB

**Figure 2:**
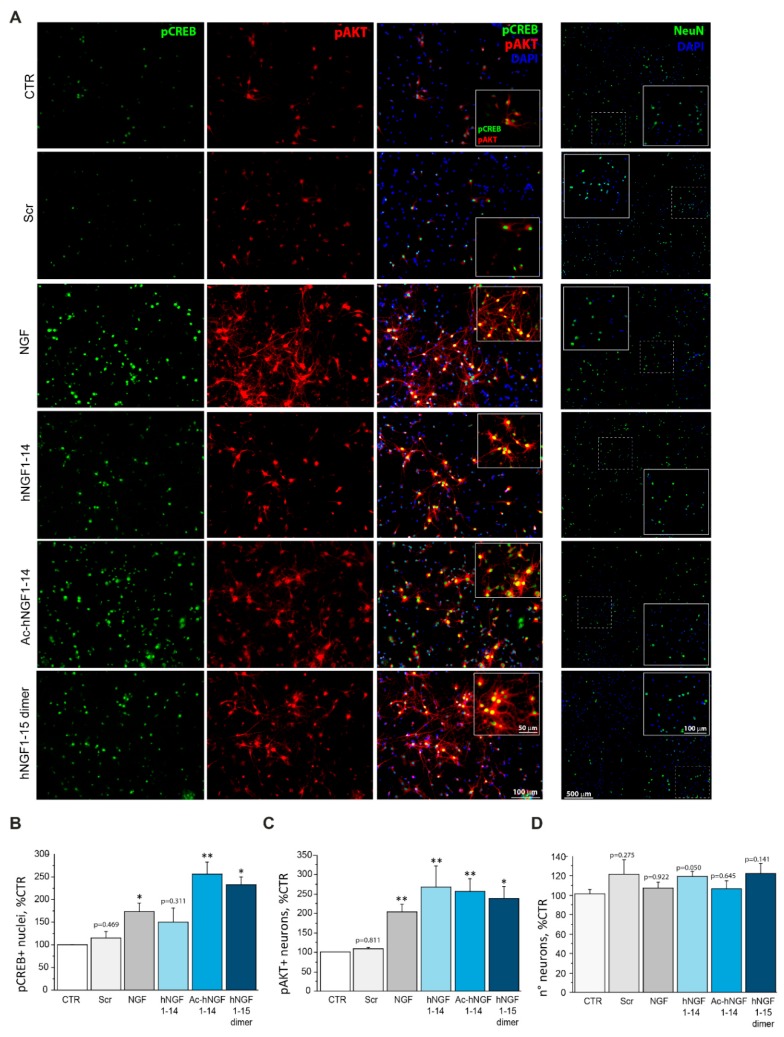
The hNGF1–14 peptides increase the number of pCREB and pAKT expressing cholinergic neurons. (**A**–**C**) Double immunolabelling and quantitative analysis of pCREB (**A**,**B**; green colour) and pAKT (**A**,**C**; red colour) single positive DIV1 cholinergic neurons per field (20× objective) following 20′ incubation with 10 μM each of scrambled hNGF1–14 (Scr), hNGF1–14, Ac-hNGF1–14, and hNGF1–15 dimer and 100 ng/mL (3.84 nM) NGF. Nuclei were counterstained with DAPI (blue colour). The pCREB and pAKT positive neurons were counted, expressed in percentage of CTR and compared. NGF, hNGF1–14, Ac-hNGF1–14, and hNGF1–15 dimer, but not the scrambled hNGF1–14 increased CREB activity over the control level. NGF and all hNGF1–14 derivatives except the scrambled peptide, were able to promote AKT phosphorylation in cholinergic neurons. Note in inlets that the majority of pCREB positive cholinergic neurons are also immunolabelled for pAKT (yellow colour), indicating a coherent activation of the survival pathway in NGF and peptides driven CREB signalling. (**A**,**D**) NeuN staining of neuronal nuclei followed by DAPI nuclear counterstaining is shown at low and high magnification (A, inlet). Further, the total number of neuronal nuclei, as seen by the marker NeuN, over the total number of DAPI positive nuclei was counted. The analysis showed that percentage neurons cultured with NGF, hNGF1–14, Ac-hNGF1–14, hNGF1–15 dimer, and scrambled NGF1–14 are comparable to control conditions. Data are from *n* = 3 independent experiments and were expressed as percentage of CTR and reported as mean +SEM. * *p* < 0.05; ** *p* < 0.01. Scale bar = 100 μm; inlets = 50 μm, 100 μm.

Then, we assessed whether hNGF1–14 peptides are able not only to activate key signalling molecules of the NGF-TrkA pathway, but also to engage an increased number of cholinergic neurons in the NGF-like pro-survival neurotrophic response. To this purpose, we incubated cholinergic neurons for 20′ either with 10 μM scrambled hNGF1–14, or 100 ng/mL (3.84 nM) NGF, or 10 μM hNGF1–14, or 10 μM Ac-hNGF1–14, or 10 μM hNGF1–15 dimer, and number of pCREB (Figure 2A,B) and pAKT (Figure 2A,C) immunolabelled neurons were counted and compared.

We observed that NGF augmented the percentage of pCREB (172.86 ± 15.60% of CTR; * *p* < 0.05) and pAKT (191.33 ± 28.04% of CTR; ** *p* < 0.01) positive cholinergic neurons (Figure 2A,B). Further, we demonstrated that Ac-hNGF1–14 (256.56 ± 14.23% of CTR; ** *p* < 0.01) and hNGF1–15 dimer (232.32 ± 29.62% of CTR; * *p* < 0.05), but not hNGF1–14 (150.47 ± 40.56% of CTR; *p* = 0.311) and scrambled hNGF1–14 (113.94 ± 7.20% of CTR; *p* = 0.469) augmented the number of pCREB positive neurons. Further, we observed that cholinergic neurons expressing pAKT were significantly increased also upon hNGF1–14 (250.39 ± 27.17% of CTR; ** *p* < 0.01), Ac-hNGF1–14 (240.23 ± 25.01% of CTR; ** *p* < 0.01), and hNGF1–15 dimer (222.91 ± 24.52% of CTR; * *p* < 0.05) incubation (Figure 2A,C). On the contrary, the scrambled hNGF1–14 (101.27 ± 3.43% of CTR; *p* = 0.811) was not able to affect the number of pAKT positive neurons, as compared to control (100.00 ± 3.59%). To ensure that increase in activated neurons is a measure of NGF or peptide stimulations, and not the result of increased number of neurons, total NeuN positive neuronal nuclei were counted and compared (Figure 2A,D). As expected, the results reported in Figure 2D show that neurons are not affected in number by treatments with NGF (104.69 ± 5.93% of CTR; *p* = 0.922), hNGF1–14 (117.00 ± 4.54% of CTR; *p* = 0.050), Ac-hNGF1–14 (103.95 ± 8.30% of CTR; *p* = 0.645), hNGF1–15 dimer (119.63 ± 10.17% of CTR; *p* = 0.141), and scrambled NGF1–14 (118.77 ± 14.77% of CTR; p = 0.275), as compared to control conditions (99.26 ± 4.51%).

Since cholinergic cultures are not pure neuronal cultures pCREB and MAP2 double immunofluorescent labellings were performed, in order to exclude any glial contribution to the neurotrophic effect. As shown in Supplementary Figure S3, a great majority of pCREB stained cells (*green* colour) also express MAP2 (red colour), pinpointing that CREB activation upon NGF or peptides incubation mainly occurs in neurons.

### 3.3. hNGF1–14 Peptides Show NGF-like Activity in Stimulating Neuronal Activity (c-Fos), Cholinergic Functions (ChAT) and Neuronal Plasticity (BDNF)

**Figure 3:**
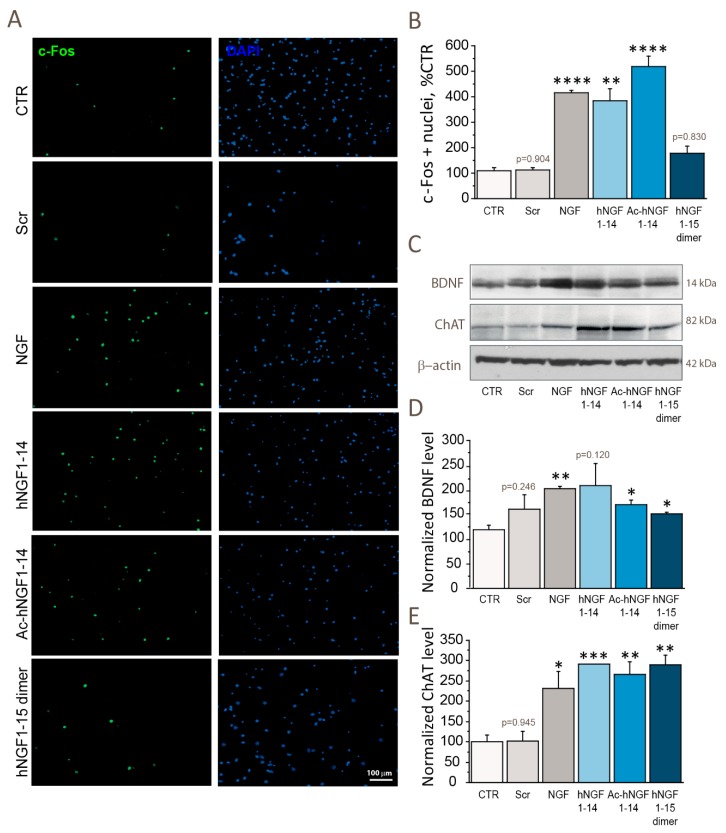
hNGF1–14 peptides mimic NGF by inducing neuronal activity, BDNF and ChAT levels. (**A**,**B**) Quantitative analysis of the ratio of c-Fos expressing nuclei in cholinergic neurons incubated for 60′ with 10 μM scrambled hNGF1–14 (Scr), 10 μM hNGF1–14, 10 μM Ac-hNGF1–14, 10 μM hNGF1–15 dimer and 100 ng/mL (3.84 nM) NGF. Neurons were immunolabelled for c-Fos and nuclei were counterstained with 4′,6-diamidino-2-phenylindole (DAPI). The number of c-Fos positive nuclei was calculated as ration over the total number of nuclei per field, and reported as percentage of CTR. (**C**, **E**) Representative western blotting (**C**) against BDNF and ChAT in cholinergic neurons incubated for 60′ with 10 μM scrambled hNGF1–14 (Scr), 10 μM hNGF1–14, 10 μM Ac-hNGF1–14, 10μM hNGF1–15 dimer and 100 ng/mL (3.84 nM) NGF. (**D**,**E**) Densitometric analysis of BDNF (**D**) and ChAT (**E**) level in cholinergic neurons treated for 60′ with 10 μM scrambled hNGF1–14 (Scr), 10 μM hNGF1–14, 10 μM Ac-hNGF1–14, 10 μM hNGF1–15 dimer and 100 ng/mL (3.84 nM) NGF. The c-Fos (*n* = 4), BDNF (*n* = 3) and ChAT (*n* = 3) data were expressed as percentage of CTR and reported as mean ± SEM. The β-actin was used as loading control. * *p* < 0.05; ** *p* < 0.01; *** *p* < 0.001; **** *p* < 0.0001. Full-length blots are presented in the Supplementary Material. Scale bar = 100 μm.

To evaluate the ability of hNGF1–14 peptides to elicit neuronal activity of cholinergic neurons, we incubated cholinergic neurons for 60′ with 10 μM scrambled hNGF1–14, or 100 ng/mL (3.84 nM) NGF, or 10 μM hNGF1–14, or 10 μM Ac-hNGF1–14, or 10 μM hNGF1–15 dimer. The number of nuclei positive to the immediate early gene (IEG) c-Fos, an established marker of cellular activation upon an acute stimulus was counted and compared among experimental groups (Figure 3A,B).

NGF incubation exerted a stimulating effect on nuclear c-Fos level in cholinergic neurons (414.24 ± 9.59% of CTR; **** *p* < 0.0001) versus control (109.44 ± 11.38%). A comparable effect was observed following treatment with hNGF1–14 (383.81 ± 46.00% of CTR; ** *p* < 0.01) and Ac-hNGF1–14 (518.39 ± 40.77% of CTR; **** *p* < 0.0001, but not with the hNGF1–15 dimer (177.18 ± 30.55% of CTR; *p* = 0.830), and scrambled hNGF1–14 (111.44 ± 10.99% of CTR; *p* = 0.904).

We also investigated the amount of mature BDNF (~14 kDa) by western blotting following NGF and hNGF1–14 peptides (Figure 3C,D). We found that BDNF level is potentiated by NGF (205.45 ± 4.41% of CTR; ** *p* < 0.01), as well as by Ac-hNGF1–14 (172.26 ± 8.38% of CTR; * *p* < 0.05), and hNGF1–15 dimer (153.28 ± 2.90% of CTR; * *p* < 0.05). No statistically significant difference was found between the hNGF1–14 (211.24 ± 45.43% of CTR; *p* = 0.120), or the scrambled hNGF1–14 (162.30 ± 29.76% of CTR; *p* = 0.246) and control neurons (119.56 ± 10.24%).

Last, we assessed the expression of the cholinergic marker ChAT and observed that NGF (231.51 ± 42.12% of CTR; * *p* < 0.05), hNGF1–14 (291.03 ± 0.60% of CTR; *** *p* < 0.001), Ac-hNGF1–14 (265.58 ± 30.75% of CTR; ** *p* < 0.01), and hNGF1–15 dimer (289.06 ± 24.62% of CTR; ** *p* < 0.01) but not scrambled hNGF1–14 (102.15 ± 23.98% of CTR; *p* = 0.945) significantly stimulate ChAT level (Figure 3C,E).

### 3.4. The Neurotrophic Effect of hNGF1–14 Peptides on Cholinergic Neurons is Lost upon TrkA Inhibition

**Figure 4:**
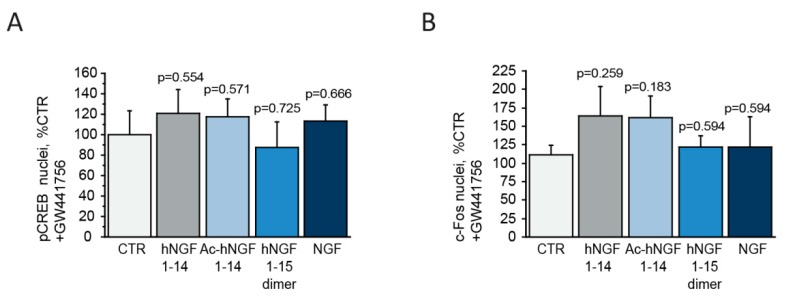
Effect of hNGF1–14 peptides on c-Fos and pCREB are abolished by a specific and potent TrkA inhibitor. (**A**,**B**) Quantitative analysis of pCREB and c-Fos positive nuclei in cholinergic neurons incubated for 60′ with 10 μM scrambled hNGF1–14 (Scr), 10 μM hNGF1–14, 10 μM Ac-hNGF1–14, 10 μM hNGF1–15 dimer and 100 ng/mL (3.84 nM) NGF following preincubation with the specific TrkA inhibitor GW441756 (15 μM, 90′). Neurons were immunostained for pCREB or c-Fos and nuclei were counterstained with 4′,6-diamidino-2-phenylindole (DAPI) and visualized by epifluorescent microscopy. The number of pCREB and c-Fos immunolabelled nuclei was calculated as ratio over the total number of DAPI positive nuclei per field (20× objective). Data are from *n* = 3 independent experiments and were expressed as percentage of CTR and reported as mean +SEM.

To assess TrkA specificity of the hNGF1–14 peptides, key experiments were repeated in presence of the TrkA inhibitor to assess TrkA requirement for the NGF pathway activation by hNGF1–14 peptides. We incubated cholinergic neurons with the potent and highly specific TrkA inhibitor GW441756 (15 μM, 90′) and then stimulated with 10 μM hNGF1–14, 10 μM Ac-hNGF1–14, 10 μM hNGF1–15 dimer or 100 ng/mL (3.84 nM) NGF. Peptides or NGF stimulation incubation lasted for 30′ for subsequent anti-pCREB (Figure 4A), or for 60′ for anti-c-Fos (Figure 4B) immunostainings. The hNGF1–14 peptides have been shown to induce an increase in the number of pCREB (Figure 2A,B) expressing neurons and c-Fos positive nuclei (Figure 3A,B), in a manner comparable to that of the whole NGF molecule. However, upon incubation of cholinergic neurons with the specific TrkA inhibitor GW441756, the hNGF1–14 peptides, as well as NGF, lack the ability to stimulate this neurotrophic response. In fact, pCREB level was comparable to control untreated cholinergic neurons (100.00 ± 23.43%) in hNGF1–14 (120.81 ± 23.55% of CTR; *p* = 0.554), Ac-hNGF1–14 (117.50 ± 17.38% of CTR, *p* = 0.571), hNGF1–15 dimer (87.36 ± 25.07% of CTR; *p* = 0.725), and NGF (113.00 ± 16.55% of CTR; *p* = 0.666) treated neurons pre-incubated with the TrkA inhibitor GW441756 (Figure 4A).

Similarly, nuclear c-Fos expression in hNGF1–14 (164.01 ± 40.24% of CTR; *p* = 0.259), Ac-hNGF1–14 (161.18 ± 30.41% of CTR; *p* = 0.183), hNGF1–15 dimer (122.45 ± 14.96% of CTR; *p* = 0.594), and NGF (122.06 ± 41.16% of CTR; *p* = 0.594) treated neurons after GW441756 pre-treatment was comparable to control untreated 111.21 ± 13.23%) neurons (Figure 4B).

Efficacy of the TrkA inhibitor GW441756 was verified by analysis of TrkA (Y490) phosphorylation upon cholinergic neurons incubation with GW441756 (15 μM, 90′) followed by stimulation with hNGF1–14 (10 μM, 10′), Ac-hNGF1–14 (10 μM, 10′), hNGF1–15 dimer (10 μM, 10′), and NGF (100 ng/mL; 3.84 nM, 10′) as positive control. Preincubation with GW441756 inhibited TrkA activation by NGF, as well as by hNGF1–14 peptides (Supplementary Figure S2C,D).

### 3.5. Ac-hNGF1–14 Peptide Administration Induces an NGF-like Electrophysiological Activity in Primary Cholinergic Neurons

To verify the status of excitatory neurotransmission in cholinergic neurons exposed to Ac-hNGF1–14 peptide, we have studied the miniature excitatory post synaptic currents (mEPSCs) in DIV10 control exposed or not to Ac-hNGF1–14 peptide and NGF.

**Figure 5:**
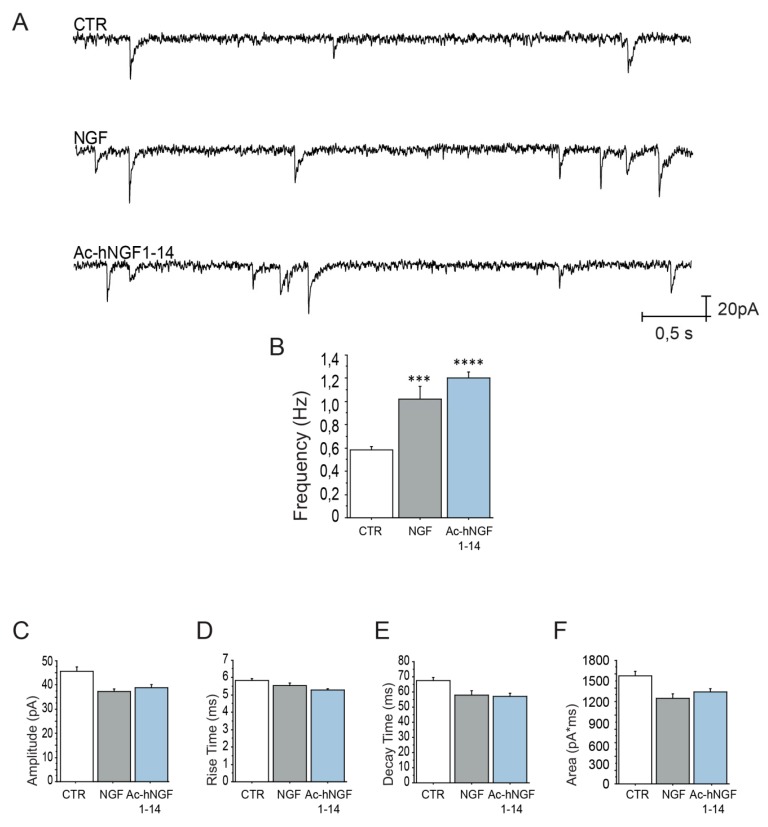
Ac-hNGF1–14 potentiates the spontaneous excitatory neurotransmission of cholinergic neurons in a NGF-like manner. (**A**) Representative traces of mEPSCs (0.5 s, 20 pA) recorded from cholinergic neurons cultures at 10 DIV in control conditions and following 30 min treatment (37 °C) with NGF (100 ng/mL; 3.84 nM) and Ac-hNGF1–14 peptide (10 µM), respectively. (**B**–**F**) Bar plots reporting the mean ±SEM frequency (**B**), amplitude (**C**), rise time (**D**), decay time (**E**) and area (**F**) of the mEPSCs recorded in the three experimental conditions. Neurons incubated with NGF (NGF1.08 ± 0.1 Hz; *** *p* < 0.001) and Ac-hNGF1–14 peptide (1.2 ± 0.01 Hz; **** *p* < 0.0001) show a mEPSC frequency significantly higher than control neurons (CTR, 0.58 ± 0.05 Hz). Significant differences were not detected for the other analysed parameters, like amplitude (CTR: 45.9 + 2.84 pA; NGF: 39.1 + 2.4 pA; *p* = 0.18; Ac-hNGF1–14: 37.4 + 2.2 pA, *p* = 0.07), rise time (CTR: 5.82 + 0.2 ms; NGF: 5.21 + 0.2 ms, *p* = 0.24, Ac-hNGF1–14: 5.53 + 0.3 ms, *p* = 1.00), decay time (CTR: 67.6 + 3.12 ms; NGF: 57.22 + 3.4 ms, *p* = 0.18; Ac-hNGF1–14: 58.11 + 4.9 ms, *p* = 0.27), and area (CTR: 1576 + 119.4 ms*pA; NGF: 1337 + 87.15ms*pA; *p* = 0.36; Ac-hNGF1–14:1246 + 116.7ms*pA; *p* = 0.11), as compared to control neurons (CTR). *** *p* < 0.001; **** *p* < 0.0001. Data (*n* = 19 neurons for CTR and NGF; *n* = 20 neurons for Ac-hNGF1–14) were from 3 different experiments.

Figure 5A shows mEPSCs in representative cholinergic neurons in control condition (CTR), following incubation at 37 °C with NGF (NGF; 100 ng/mL; 3.84 nM, 30‘) or Ac-hNGF1–14 peptide (Ac-hNGF1–14; 10 µM, 30‘). The analysis of the mEPSCs has confirmed the previously reported NGF-induced potentiation of excitatory neurotransmission in these neurons [45,46]. In fact, cholinergic neurons exposed to NGF (1.08 ± 0.1 Hz; *** *p* < 0.001) showed mEPSC frequency (Figure 5B) significantly higher than control (0.58 ± 0.05 Hz). Interestingly, also neurons exposed to the Ac-hNGF1–14 peptide showed a mEPSC frequency significantly higher than CTR (1.2 ± 0.01 Hz, **** *p* < 0.0001), and comparable to that measured in neurons exposed to NGF (Ac-hNGF1–14 versus NGF: *p* = 0.930; *n* = 19), suggesting a presynaptic neurotransmitter release facilitation induced by both NGF and hNGF1–14 peptide.

As control for the peptide sequence specificity and for TrkA-dependent NGF effect, the electrophysiological activity of scrambled hNGF1–14 (Scr; 10 µM, 30‘) and of NGF (NGF; 100 ng/mL; 3.84 nM, 30‘) in presence of the TrkA inhibitor GW441756 (GW441756 + NGF; 10 µM, 45‘) were analysed in another set of experiments. Any statistical significance was found for mEPSC frequency (CTR: 0.58 + 0.07 Hz; Scr: 0.63 + 0.07 Hz; *p* = 1; GW441756 + NGF: 0.65 + 0.06 Hz; *p* = 1), amplitude (CTR: 42.58 + 3.18 pA; Scr; 41.4 + 3.1 Hz; *p* = 1; GW441756 + NGF: 39.8 + 2.4 pA; *p* = 1), rise time (CTR: 6.37 + 0.4 ms; Scr: 6.44 + 0.4 ms; *p* = 1; GW441756+NGF: 6.64 + 0.4 ms; *p* = 1.00), decay time (CTR: 75.5 + 4.5 ms; Scr: 72.6 + 5.3 ms; *p* = 1; GW441756 + NGF: 73.8+4.8 ms; *p* = 1), and area (CTR: 1280 + 89.7 ms*pA; Scr: 1106 + 121.4 ms*pA; *p* = 0.84; GW441756 + NGF: 1265 + 118.9 ms*pA; *p* = 0.1 vs. CTR; p0.95 vs. Scr).

### 3.6. hNGF1–14 Peptides Sustain the Survival and Promote Neurite Outgrowth of Dissociated Dorsal Root Ganglia Neurons

**Figure 6:**
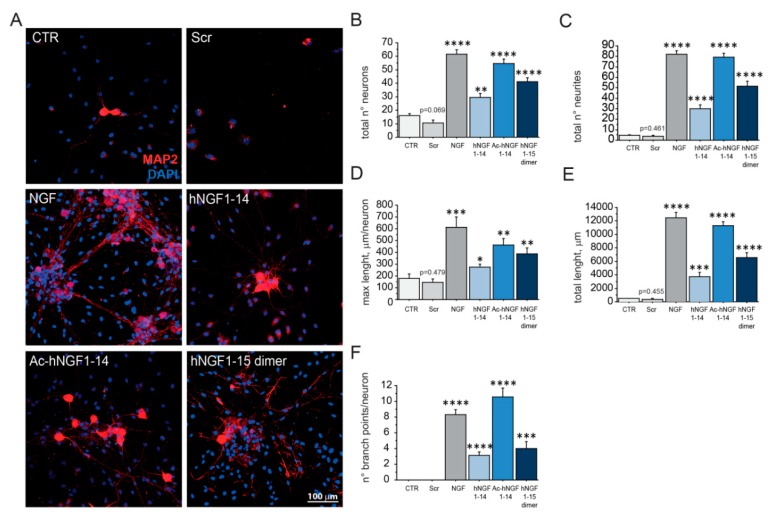
hNGF1–14 peptides sustain survival and neurite elongation of DRG dissociated neurons in absence of NGF. (A-F) Classical DRG assay to test NGF-like bioactivity of hNGF1–14 peptides. (**A**) Dissociated DRG neurons plated and cultured for 5 days in presence of hNGF1–14 peptides (10 μM) or NGF (100 ng/mL; 3.84 nM). DRG neurons were fixed, immunolabelled with anti-MAP2 antibody for neurites staining, and nuclei were counterstained with DAPI. Images were acquired by means of confocal microscopy. Scrambled hNGF1–14 treated and untreated (CTR) DRG neurons survived although in lower number, and lacked of axons and neurites, typical of fully differentiated DRG neurons, as seen by absence of MAP2 staining (red colour). DRG neurons cultured with 10 μM hNGF1–14, 10 μM Ac-hNGF1–14, 10 μM hNGF1–15 dimer exhibit a mature DRG phenotype, including MAP2 positive neurite extension and an extensive neuronal network, resembling that promoted by the entire NGF molecule (NGF; 100 ng/mL; 3.84 nM, F). (**B**–**F**) DRG neurons survival and neurite outgrowth were analysed and quantified on MAP2 stained DIV5 DRG neurons by means of several parameters. (**B**) Number of surviving neurons: CTR (16.00 ± 1.58%), Scr (10.43 ± 2.30% of CTR; *p* = 0.069), NGF (122.06 ± 41.16% of CTR; **** *p* < 0.0001), hNGF1–14 (29.57 ± 2.85% of CTR; ** *p* < 0.01), Ac-hNGF1–14 (54.71 ± 3.41% of CTR; **** *p* < 0.0001), hNGF1–15 dimer (41.84 ± 2.94% of CTR; **** *p* < 0.0001). (**C**) Number of total neurites per field: CTR (4.57 ± 0.5%), Scr (3.71 ± 1.02; *p* = 0.461), NGF (82.14 ± 2.96; **** *p* < 0.0001), hNGF1–14 (29.86 ± 3.71; **** *p* < 0.0001), Ac-hNGF1–14 (79.00 ± 4.17; **** *p* < 0.0001), hNGF1–15 dimer (51.71 ± 4.71; **** *p* < 0.0001). (**D**) The maximum neurite length (length of the longest neuritis): CTR (180.87 ± 35.76 μm), Scr (146.67 ± 630.13 μm; *p* = 0.479), NGF (612.66 ± 87.60 μm; *** *p* < 0.001), hNGF1–14 (276.22 ± 23.87 μm; * *p* < 0.05), Ac-hNGF1–14 (460.92 ± 55.07 μm; ** *p* < 0.01), hNGF1–15 dimer (385.51 ± 52.61 μm; ** *p* < 0.01). (**E**) Total neurites length: CTR (479.02 ± 62.41 μm), Scr (383.86 ± 106.38 μm; *p* = 0.455), NGF (12471.32 ± 815.57 μm; **** *p* < 0.0001), hNGF1–14 (3753.74 ± 603.71 μm; *** *p* < 0.001), Ac-hNGF1–14 (11301.31 ± 548.43 μm; **** *p* < 0.0001), hNGF1–15 dimer (6586.87 ± 685.52 μm; **** *p* < 0.0001). (**F**) The number of total branch points: CTR (0), Scr (0), NGF (8.29 ± 0.64; **** *p* < 0.0001), hNGF1–14 (3.14 ± 0.40; **** *p* < 0.0001), Ac-hNGF1–14 (10.57 ± 1.13; **** *p* < 0.0001), hNGF1–15 dimer (4.00 ± 0.90; *** *p* < 0.001). Data (*n* = 7) were reported as mean +SEM. * *p* < 0.05; ** *p* < 0.01; *** *p* < 0.001, **** *p* < 0.0001. Scale bar = 100 μm.

Furthermore, to extend the biological characterization of hNGF1–14 peptides, we also studied their action in the classical DRG assay, a model system of choice to validate new NGF agonists for drug discovery [44]. Indeed, NGF is a critical survival, phenotypic and functional regulatory factor for sensory neurons during developmental and early postnatal period [47,48,49]. A continuous NGF supply is needed for dissociated DRG neurons survival and differentiation in vitro [50]. To assess the neurotrophic bioactivity of peptides, post-natal day 2 (P2) DRG neurons were cultured for 5 days in low-serum medium with hNGF1–14 peptides (10 μM) or NGF (100 ng/mL; 3.84 nM). DRG neurons cultured with NGF or hNGF1–14 peptides and MAP2 stained neurons were analysed (Figure 6).

Number of surviving neurons (Figure 6B) in NGF (122.06 ± 41.16% of CTR; **** *p* < 0.0001), hNGF1–14 (29.57 ± 2.85% of CTR; ** *p* < 0.01), Ac-hNGF1–-14 (54.71 ± 3.41% of CTR; **** *p* < 0.0001), hNGF1–15 dimer (41.84 ± 2.94% of CTR; **** *p* < 0.0001) were statistically higher than CTR (16.00 ± 1.58%). The scrambled peptide did not exert any effect (10.43 ± 2.30% of CTR; *p* = 0.069).

Assessment of total neurites per field (Figure 6C) showed that NGF (82.14 ± 2.96; **** *p* < 0.0001), hNGF1–14 (29.86 ± 3.71; **** *p* < 0.0001), Ac-hNGF1–14 (79.00 ± 4.17; **** *p* < 0.0001), hNGF1–15 dimer (51.71 ± 4.71; **** *p* < 0.0001), but not scrambled peptide (3.71 ± 1.02; *p* = 0.461), were able to promote neurite elongation, as compared to CTR (4.57 ± 0.5%). The maximum neurite length (Figure 6D) and total neurites length (Figure 6E) were also examined, and the results confirmed the neurotrophic effect of NGF (612.66 ± 87.60 μm; *** *p* < 0.001 and 12471.32 ± 815.57 μm; **** *p* < 0.0001 respectively), as well as of hNGF1–14 (276.22 ± 23.87 μm; * *p* < 0.05 and 3753.74 ± 603.71 μm; *** *p* < 0.001, respectively), Ac-hNGF1–14 (460.92 ± 55.07 μm; ** *p* < 0.01 and 11301.31 ± 548.43 μm; **** *p* < 0.0001, respectively), hNGF1–15 dimer (385.51 ± 52.61 μm; ** *p* < 0.01 and 6586.87 ± 685.52 μm; **** *p* < 0.0001, respectively) and comparable to, as compared to control CTR (180.87 ± 35.76 μm and 479.02 ± 62.41 μm, respectively). Scrambled peptide did not exert any neurotrophic effect (146.67 ± 630.13 μm; *p* = 0.479 and 383.86 ± 106.38 μm; *p* = 0.455, respectively). Also, the number of total branch points was evaluated (Figure 6F), as a measure of DRG network formation in NGF (8.29 ± 0.64; **** *p* < 0.0001), hNGF1–14 (3.14 ± 0.40; **** *p* < 0.0001), Ac-hNGF1–14 (10.57 ± 1.13; **** *p* < 0.0001), and hNGF1–15 dimer (4.00 ± 0.90; *** *p* < 0.001). Notably, the number of branch points in the control condition and upon scrambled peptide incubation was equal to zero. The scrambled hNGF1–14 did not exert any neurotrophic action, resulting in lack of neurite elongation and reduced survival, with a trend comparable to control.

## 4. Discussion

The aim of this study was to evaluate in vitro efficacy of hNGF1–14 and its derivatives as potential drugs for in vivo neuroprotection, of interest for novel therapeutic approaches in neurodegenerative pathologies. Given the limitation of immortalized cell lines studies, to test NGF mimetic activity of the hNGF1–14 sequence we resorted to two types of well-known NGF-target primary neurons, as cellular paradigm of choice.

In this study, we investigated and compared the hNGF1–14, its monomeric derivative peptide Ac-hNGF1–14, and the hNGF1–15 dimer, using NGF as positive and a scrambled peptide as negative controls. Here, we report that the Ac-hNGF1–14, hNGF1–14 and, to some extent, the hNGF1–15 dimer trigger a TrkA-dependent NGF-like signalling cascade in cholinergic neurons (Supplementary Figure S1E) and show survival and neuritogenic properties in dissociated DRG neurons.

The rationale for using the N terminal sequence of the human NGF is mainly based on the biological implication of the N terminal domain for NGF-TrkA binding and pathway activation [51,52,53]. Indeed, it has been previously shown that hNGF1-14 is able, in fact, to bind TrkA through several non-covalent interactions mainly with the TrkA-D5 domain [28,36]. In line with this, the N-terminal human sequence 1–14 (hNGF1–14) and its acetylated derivative Ac-hNGF1–14 demonstrated a good NGF-like in vitro activity by protecting PC12 cells from NGF withdrawal driven degeneration [27] as well as neurotrophic signalling in PC12 and neuroblastoma cell lines [27,36]. Recently, the hNGF1–15 dimer has been shown to have CREB-dependent neurotrophic activities in PC12 [28].

To deeply assess their ability to stimulate NGF/TrkA signalling and NGF typical bioactivity, we resorted to two elective cellular models of NGF-target neurons of the central (CNS) and peripheral (PNS) nervous systems, the cholinergic and DRG primary neurons, respectively. The cultured cholinergic neurons do not need NGF to survive, thus being a good paradigm to test the short-term effect of an acute NGF stimulus, e.g., signalling pathway and neuronal activity. Opposite, since DRG neurons require NGF/TrkA continuous supply to survive and differentiate, they represent the model of choice for in vitro survival assays with putative NGF mimetics.

Here we show that the monomeric (hNGF1–14) acetylated monomeric (Ac-hNGF1–14) and dimeric hNGF1–15 peptides present NGF typical signalling properties, being able to stimulate its specific receptor TrkA, its early adaptors ShcC and PLC-γ, as well as its downstream PI3K and MAPK signalling molecules, finally leading to activation of survival promoting AKT pathways (Figure 1). Taken together, these findings suggest that the TrkA pathway initiated by hNGF1–14 peptides lead to convergence of Shc and PLC-γ neurotrophic signalling on key downstream molecules and cellular endpoints in cholinergic neurons, resembling the NGF prototypical mechanism of action.

Note worthily, 10 μM Ac-hNGF1–14 incubation resulted in a statistically significant phosphorylation increase of ShcC (~200%; *p* < 0.01), PI3K (*p* < 0.01) and AKT (*p* < 0.01), as compared to the optimal NGF dose (100 ng/mL; 3.84 nM). Also, we demonstrated that hNGF1–14 peptides mimic NGF in engaging an increased number of neurons responding by activation of key signalling intermediates, like CREB and AKT (Figure 2A–C), without affecting number of total neuron (Figure 2D).

The findings described herein pinpoint a consistent neurotrophic effect of the acetylated derivative, in agreement with the highest number of non-covalent interactions occurring between Ac-hNGF1–14 and the D5 domain of TrkA in comparison with the other peptides derivatives [36]. Furthermore, it has been reported that only hNGF(1–14), but not its reverse sequence hNGF(14–1) nor the scrambled hNGF(1–14) sequence, has strong and significant interactions with the D5 domain of the TrkA receptor [28], further pinpointing the sequence dependency of the natural human NGF sequence of the hNGF1–14 peptide. We used 10 μM as optimal hNGF1–14 in vitro dose in this study, based on the preliminary dose-response viability test (Supplementary Figure S1), on dose-dependent TrkA activation in cholinergic neurons (Supplementary Figure S2A), and on DRG assay (Supplementary Figure S2B). The two dose-response analyses demonstrate that hNGF1–14 peptides are not toxic to neurons and induce TrkA activation in the 2–10 μM range. Herein, the 10 μM has been used as the concentration of choice, as it was already done in previous works with PC12 and neuroblastoma [27,28,36]. In line, TrkA agonists effective in the micromolar range have been already reported by other studies [12,54].

Albeit glial cells are a minor component of embryonic septal cultures (less than 5% total cells), [45] and low density plating used in this study is sufficient *per se* to minimize glial survival and growth, a glial contribution to hNGF1–-14 peptides-driven NGF pathway activation cannot be excluded. To address this question, we performed double pCREB/MAP2 immunofluorescence labelling. As reported in Supplementary Figure S3, we found that all pCREB cells are also MAP2 positive in control conditions as well as upon NGF/hNGF1–14 peptides incubation, indicating that pCREB expressions upon NGF/peptides *stimuli* occur mainly in neurons (~85% of cells). Our data are in line with previous works indicating that NGF specifically affects cholinergic neurons in culture [45]. Overall, glial targeting by hNGF1–14 peptides in cholinergic cultures may be rule out.

As a result of CREB activation, a crucial hub in neurotrophic responses [55], the hNGF1–14 peptides showed the ability to increase neuronal activity, measured by nuclear c-Fos level (Figure 3A,B). The c-Fos is an early immediate gene, induced by neuronal depolarization. For this reason, it is commonly considered a marker for neuronal activity both in vitro and in vivo [56,57]. The observed increase of mature BDNF (Figure 3C,D) and ChAT (Figure 3C,E) levels indicated a coherent stimulation of the synaptic neurotrophin and of Ach metabolism. Noteworthy, CREB has been suggested to have a pivotal role in NGF-driven c-Fos promotion [58,59], leading to subsequent BDNF [60], and ChAT [61] expressions in neurons.

Further, our findings are in line with the previously reported increase of BDNF mRNA level and BDNF release upon hNGF1–14 peptides incubation of PC12 cells [28,36]. Of note, reduced ChAT expression deficits occur in several age-related CNS pathologies, like AD [62] and PD [63] and in neurodevelopmental disorders [64] even in absence of neuronal death. Moreover, BDNF is recognized as a key neurotrophin modulating forebrain LTP and synaptic plasticity [65], and its expression is induced by NGF in target neurons [66]. Thus, the use of hNGF1–14 peptides to sustain BDNF and ChAT levels is of interest for several human diseases associated to cholinergic disturbances, like AD, PD, DS and cancer.

Interestingly enough, the peptidomimetics property of hNGF1–14 peptides was abolished by previous incubation with the TrkA specific inhibitor GW441756 (15 μM, 90′). As compared to other general tyrosine kinase receptors inhibitors used so far, like K252a and synthetic derivatives (e.g., CEP), the GW441756 is a potent TrkA inhibitor (15 μM, IC_50_ = 2 nM) with more than 100-fold selectivity over other kinases, thus allowing the highly specific targeting of the NGF/TrkA pathway. The lack of hNGF1–14 effect in presence of the inhibitor strongly suggests that an active TrkA receptor is required for its CREB/c-Fos related neurotrophic action in cultured cholinergic neurons.

This study was mainly focused on the ability of hNGF1–14 peptides to activate the NGF specific TrkA receptor. Other receptors expressed by cholinergic neurons, like p75 common neurotrophic receptor and the specific BDNF receptor TrkB, were not addressed. However, the crystal structure analysis of the NGF–TrkA-IgGL2 complex clearly showed that N-terminal residues are dispensable for NGF interaction with the p75 receptor [26], while p75-NGF binding occurs via the L1, L3 and L4 loops [16], and the D1 domain in presence of TrkA [22,26]. Also, it has been previously observed that hNGF1–14 is able to promote activation upon endocytosis of TrkA, but not of p75NTR in neuronal cells [28,36]. Cumulatively, and given the observed neurotrophic outcome of hNGF1–14 peptides in TrkA-expressing cholinergic neurons, the induction of the p75-driven signalling by 10 μM hNGF1–14 peptides may be excluded. Whether the peptides are able to activate a p75NTR homodimers apoptotic signalling [67] in absence of TrkA remains to be elucidated.

As for hNGF1–14 peptides interplay with the BDNF pathway, crystallization studies of the peptides complexed with the TrkB-D5 domain are not yet available. Based on current knowledge, human BDNF lacks the two histidines critical for TrkA binding at its N-terminus, and it was reported not to bind TrkA-D5, further suggesting neurotrophins specificity at their N-terminal region [32,33]. In line, cross-interactions of hNGF1-14 peptides with other neurotrophic pathways are seemingly unlike in these cellular models. Nonetheless, this critical issue deserves further scrutiny in preclinical testings of hNGF peptides.

Increased NGF availability has been reported to significantly facilitate the induction of hippocampal long-term potentiation (LTP) [68], by augmenting the frequency of mEPSCs in the cholinergic system [46,69]. Thus, the electrophysiological functions of Ac-hNGF1–14, the peptide with the highest in vitro activity, were assessed. We found that 10 μM Ac-hNGF1–14 induces a facilitation of presynaptic excitatory neurotransmitter release (Figure 5), and exerts a potent and specific stimulatory presynaptic action on cholinergic nerve terminals [46,69] similarly to 3.84 nM NGF holoprotein [45,70]. According to the literature, NGF-induced neurotransmitter release potentiation was found to occur upon a significant mEPSC frequency elevation (Figure 5B), and a comparable effect was found following incubation with the Ac-hNGF1–14 peptide (Figure 5B) in cholinergic cultures. As control for the peptide sequence specificity and for TrkA-dependent NGF effect, the electrophysiological activity of scrambled hNGF1–14 (Scr; 10 µM, 30‘) and of NGF (NGF; 100 ng/mL; 3.84 nM, 30‘) in presence of the TrkA inhibitor GW441756 (GW441756 + NGF; 10 µM, 45‘) were analysed in another set of experiments. Any statistical significance was found for mEPSC parameters examined, like frequency, amplitude, rise time, decay time, and area (Supplementary Figure S4A,B).

Since TrkA activation is known to occur by NGF dimers binding, dimeric or cyclised peptidomimetics have been generally preferred to facilitate TrkA conformational rearrangement and subsequent autophosphorylation [71,72]. Nonetheless, major limitations have been often reported for dimeric compounds because of their partial TrkA agonism or even antagonism [73].

Interestingly enough, peptidic as well as non-peptidic monomeric compounds, like D3, have been shown to induce TrkA dimerization and subsequent DRG differentiation [12]. The exact molecular mechanisms underlying this activation need to be elucidated. However, peptides clustering over the receptor accounting for its dimerization, has been proposed at least in vitro [28].

Taking all these findings into account, preferring primary neurons to test hNGF1–14 peptides allowed full disclosure of their NGF-mimetic potential. At variance with studies performed on PC12 cell lines [27,36], micromolar concentrations of hNGF1–14 peptides were able to sustain neuronal survival and differentiation in dissociated DRG neurons (Figure 6), and ERK/MAPK activation in cholinergic neurons (Figure 1F), at an extent comparable with the NGF optimal dose. A possible reason for the discrepancies may relies on the distinct signalling machineries, activation thresholds and even differentiative status characteristics of PC12 derived neuron-like cells and primary neurons. In particular, the need of PC12 for a sustained TrkA activation because of the very low ERK/MAPK basal level during their first differentiation week may account for the suboptimal hNGF1-14 effect observed in PC12 cells [51]. In line with this, the higher ERK level in differentiated PC12 [51] allowed its activation by hNGF1–14 peptide-driven phosphorylation [36], as well as neuroprotection upon NGF withdrawal [27].

Overall, the findings here reported establish hNGF1–14 sequence as a linear monomeric TrkA agonist with full NGF-like bioactivity on NGF-target neurons. Further assessment of their efficacy as neuroprotective drugs in animal models of human CNS neurodegenerative pathologies is underway.

## 5. Conclusions

The drawbacks and limitations of previous clinical trials and NGF-based therapeutic approaches also in recent times, paved the way for the screening of novel, potent and selective NGF mimetics. Nowadays, the characterization of novel small peptidomimetic is taking centre stage in order to overcome poor oral NGF bioavailability, size-related limitations of NGF brain delivery, as well as pain related side-effects of peripheral NGF administration for spinal cord injury or diabetic neuroinflammation. The use of small peptidic molecules or non-peptidic compounds with NGF activity is instrumental to non-invasive brain treatment by means of nasal [74,75] and ocular [76,77] administrations, bypassing BBB and avoiding side effects (e.g., pain) typical of systemic NGF administration.

## Supplementary Materials

The following are available online at https://www.mdpi.com/2218-273X/10/2/216/s1, Figure S1: Dose-dependent effect of hNGF1–14 peptides on survival of cholinergic neurons, Figure S2: Dose-dependent effect of hNGF1–14 peptide on cholinergic and DRG neurons. TrkA inhibition by GW441756 upon hNGF1–14 and NGF treatments, Figure S3: Representative pCREB/MAP2 double immunofluorescent staining of dissociated DRG neurons cultured with NGF or hNGF1-14 peptides, Figure S4: The electrophysiological response of DIV1 cholinergic neurons to NGF is lost upon TrkA inhibition with GW441756. Any effect is observed following treatment with the scrambled peptide.

## References

[1] Levi-Montalcini R. (1987). The nerve growth factor 35 years later. Science.

[2] Sofroniew M.V., Howe C.L., Mobley W.C. (2001). Nerve growth factor signaling, neuroprotection, and neural repair. Annu. Rev. Neurosci..

[3] Pearson G., Robinson F., Beers Gibson T., Xu B., Karandikar M., Berman K., Cobb M.H. (2001). Mitogen-Activated Protein (MAP) Kinase Pathways: Regulation and Physiological Functions. Endocr. Rev..

[4] Huang E.J., Reichardt L.F. (2003). Trk Receptors: Roles in Neuronal Signal Transduction. Annu. Rev. Biochem..

[5] Gibbs R.B., Martynowski C. (1997). Nerve growth factor induces Fos-like immunoreactivity within identified cholinergic neurons in the adult rat basal forebrain. Brain Res..

[6] Bradshaw R.A., Pundavela J., Biarc J., Chalkley R.J., Burlingame A.L., Hondermarck H. (2015). NGF and ProNGF: Regulation of neuronal and neoplastic responses through receptor signaling. Adv. Biol. Regul..

[7] Chen L.W., Yung K.K.L., Chan Y.S., Shum D.K.Y., Bolam J.P. (2008). The proNGF-p75NTR-sortilin signalling complex as new target for the therapeutic treatment of Parkinson’s disease. CNS Neurol. Disord. Drug Targets.

[8] Faiq M.A., Wollstein G., Schuman J.S., Chan K.C. (2019). Cholinergic nervous system and glaucoma: From basic science to clinical applications. Prog. Retin. Eye Res..

[9] Fischer W., Wictorin K., Björklund A., Williams L.R., Varon S., Gage F.H. (1987). Amelioration of cholinergic neuron atrophy and spatial memory impairment in aged rats by nerve growth factor. Nature.

[10] Kromer L.F. (1987). Nerve growth factor treatment after brain injury prevents neuronal death. Science.

[11] Longo F.M., Manthorpe M., Xie Y.M., Varon S. (1997). Synthetic NGF peptide derivatives prevent neuronal death via a p75 receptor-dependent mechanism. J. Neurosci. Res..

[12] Maliartchouk S., Feng Y., Ivanisevic L., Debeir T., Cuello A.C., Burgess K., Saragovi H.U. (2000). A designed peptidomimetic agonistic ligand of TrkA nerve growth factor receptors. Mol. Pharmacol..

[13] Peleshok J., Saragovi H.U. (2006). Functional mimetics of neurotrophins and their receptors. Biochem. Soc. Trans..

[14] McDonald N.Q., Lapatto R., Murray-Rust J., Gunning J., Wlodawer A., Blundell T.L. (1991). New protein fold revealed by a 2.3-A resolution crystal structure of nerve growth factor. Nature.

[15] McDonald N.Q., Chao M. (1995). V Structural determinants of neurotrophin action. J. Biol. Chem..

[16] Woo S.B., Timm D.E., Neet K.E. (1995). Alteration of NH2-terminal residues of nerve growth factor affects activity and Trk binding without affecting stability or conformation. J. Biol. Chem..

[17] Krüttgen A., Heymach J.V., Kahle P.J., Shooter E.M. (1997). The role of the nerve growth factor carboxyl terminus in receptor binding and conformational stability. J. Biol. Chem..

[18] Kullander K., Kaplan D., Ebendal T. (1997). Two restricted sites on the surface of the nerve growth factor molecule independently determine specific TrkA receptor binding and activation. J. Biol. Chem..

[19] Rydén M., Ibáñez C.F. (1997). A second determinant of binding to the p75 neurotrophin receptor revealed by alanine-scanning mutagenesis of a conserved loop in nerve growth factor. J. Biol. Chem..

[20] Bradshaw R.A., Murray-Rust J., Blundell T.L., Mcdonald N.Q., Lapatto R., Ibáñez C.F. (1994). Nerve growth factor: Structure/function relationships. Protein Sci..

[21] Urfer R., Tsoulfas P., O’Connell L., Hongo J.A., Zhao W., Presta L.G. (1998). High resolution mapping of the binding site of TrkA for nerve growth factor and TrkC for neurotrophin-3 on the second immunoglobulin-like domain of the Trk receptors. J. Biol. Chem..

[22] Ibáñez C.F., Ebendal T., Barbany G., Murray-Rust J., Blundell T.L., Persson H. (1992). Disruption of the low affinity receptor-binding site in NGF allows neuronal survival and differentiation by binding to the trk gene product. Cell.

[23] Kahle P., Burton L.E., Schmelzer C.H., Hertel C. (1992). The amino terminus of nerve growth factor is involved in the interaction with the receptor tyrosine kinase p140trkA. J. Biol. Chem..

[24] Drinkwater C.C., Barker P.A., Suter U., Shooter E.M. (1993). The carboxyl terminus of nerve growth factor is required for biological activity. J. Biol. Chem..

[25] Shih A., Laramee G.R., Schmelzer C.H., Burton L.E., Winslow J.W. (1994). Mutagenesis identifies amino-terminal residues of nerve growth factor necessary for Trk receptor binding and biological activity. J. Biol. Chem..

[26] Wiesmann C., Ultsch M.H., Bass S.H., de Vos A.M. (1999). Crystal structure of nerve growth factor in complex with the ligand-binding domain of the TrkA receptor. Nature.

[27] Travaglia A., Pietropaolo A., Di Martino R., Nicoletti V.G., La Mendola D., Calissano P., Rizzarelli E. (2015). A small linear peptide encompassing the NGF N-terminus partly mimics the biological activities of the entire neurotrophin in PC12 cells. ACS Chem. Neurosci..

[28] Naletova I., Satriano C., Pietropaolo A., Gianì F., Pandini G., Triaca V., Amadoro G., Latina V., Calissano P., Travaglia A. (2019). The Copper(II)-Assisted Connection between NGF and BDNF by Means of Nerve Growth Factor-Mimicking Short Peptides. Cells.

[29] Pérez P., Coll P.M., Hempstead B.L., Martín-Zanca D., Chao M. (1995). V NGF binding to the trk tyrosine kinase receptor requires the extracellular immunoglobulin-like domains. Mol. Cell. Neurosci..

[30] Urfer R., Tsoulfas P., O’Connell L., Shelton D.L., Parada L.F., Presta L.G. (1995). An immunoglobulin-like domain determines the specificity of neurotrophin receptors. EMBO J..

[31] Wehrman T., He X., Raab B., Dukipatti A., Blau H., Garcia K.C. (2007). Structural and Mechanistic Insights into Nerve Growth Factor Interactions with the TrkA and p75 Receptors. Neuron.

[32] Banfield M.J., Naylor R.L., Robertson A.G., Allen S.J., Dawbarn D., Brady R.L. (2001). Specificity in Trk Receptor:Neurotrophin Interactions. Structure.

[33] Robertson A.G.S., Allen S.J., Mason G.G.F., Tyler S.J., Naylor R.L., Dawbarn D., Banfield M.J., Dando J.A., Clarke A.R., Brady R.L. (2001). Identification and structure of the nerve growth factor binding site on TrkA. Biochem. Biophys. Res. Commun..

[34] Ivanisevic L., Zheng W.H., Woo S.B., Neet K.E., Saragovi H.U. (2007). TrkA receptor “hot spots” for binding of NT-3 as a heterologous ligand. J. Biol. Chem..

[35] Dawbarn D., Fahey M., Watson J., Tyler S., Shoemark D., Sessions R., Zhang R., Brady L., Willis C., Allen S.J. (2006). NGF receptor TrkAd5: therapeutic agent and drug design target. Biochem. Soc. Trans..

[36] Pandini G., Satriano C., Pietropaolo A., Gianì F., Travaglia A., La Mendola D., Nicoletti V.G., Rizzarelli E. (2016). The Inorganic Side of NGF: Copper(II) and Zinc(II) Affect the NGF Mimicking Signaling of the N-Terminus Peptides Encompassing the Recognition Domain of TrkA Receptor. Front. Neurosci..

[37] Travaglia A., Arena G., Fattorusso R., Isernia C., La Mendola D., Malgieri G., Nicoletti V.G., Rizzarelli E. (2011). The inorganic perspective of nerve growth factor: interactions of Cu^2+^ and Zn^2+^ with the N-terminus fragment of nerve growth factor encompassing the recognition domain of the TrkA receptor. Chemistry.

[38] Bocchini V., Angeletti P. (1969). The nerve growth factor: purification as a 30,000-molecular-weight protein. Proc. Natl. Acad. Sci. USA.

[39] Triaca V., Sposato V., Bolasco G., Ciotti M.T., Pelicci P., Bruni A.C., Cupidi C., Maletta R., Feligioni M., Nisticò R. (2016). NGF controls APP cleavage by downregulating APP phosphorylation at Thr668: relevance for Alzheimer’s disease. Aging Cell.

[40] Sposato V., Canu N., Fico E., Fusco S., Bolasco G., Ciotti M.T., Spinelli M., Mercanti D., Grassi C., Triaca V. (2018). The Medial Septum Is Insulin Resistant in the AD Presymptomatic Phase: Rescue by Nerve Growth Factor-Driven IRS1 Activation. Mol. Neurobiol..

[41] Bonnington J.K., McNaughton P.A. (2003). Signalling pathways involved in the sensitisation of mouse nociceptive neurones by nerve growth factor. J. Physiol..

[42] Taneda K., Tominaga M., Tengara S., Ogawa H., Takamori K. (2010). Neurotropin inhibits both capsaicin-induced substance P release and nerve growth factor-induced neurite outgrowth in cultured rat dorsal root ganglion neurones. Clin. Exp. Dermatol..

[43] Janes K.A. (2015). An analysis of critical factors for quantitative immunoblotting. Sci. Signal..

[44] Melli G., Höke A. (2009). Dorsal root ganglia sensory neuronal cultures: a tool for drug discovery for peripheral neuropathies. Expert Opin. Drug Discov..

[45] Hartikka J., Hefti F. (1988). Development of septal cholinergic neurons in culture: plating density and glial cells modulate effects of NGF on survival, fiber growth, and expression of transmitter-specific enzymes. J. Neurosci..

[46] Huh C.Y.L., Danik M., Manseau F., Trudeau L.-E., Williams S. (2008). Chronic Exposure to Nerve Growth Factor Increases Acetylcholine and Glutamate Release from Cholinergic Neurons of the Rat Medial Septum and Diagonal Band of Broca via Mechanisms Mediated by p75NTR. J. Neurosci..

[47] Barde Y.A., Edgar D., Thoenen H. (1980). Sensory neurons in culture: changing requirements for survival factors during embryonic development. Proc. Natl. Acad. Sci. USA.

[48] Yip H.K., Rich K.M., Lampe P.A., Johnson E.M. (1984). The effects of nerve growth factor and its antiserum on the postnatal development and survival after injury of sensory neurons in rat dorsal root ganglia. J. Neurosci..

[49] Lee S.E., Shen H., Taglialatela G., Chung J.M., Chung K. (1998). Expression of nerve growth factor in the dorsal root ganglion after peripheral nerve injury. Brain Res..

[50] Eichler M.E., Rich K.M. (1989). Death of sensory ganglion neurons after acute withdrawal of nerve growth factor in dissociated cell cultures. Brain Res..

[51] Chang J.H., Mellon E., Schanen N.C., Twiss J.L. (2003). Persistent TrkA Activity Is Necessary to Maintain Transcription in Neuronally Differentiated PC12 Cells. J. Biol. Chem..

[52] Berrera M., Cattaneo A., Carloni P. (2006). Molecular simulation of the binding of nerve growth factor peptide mimics to the receptor tyrosine kinase A. Biophys. J..

[53] Longo F.M., Massa S.M. (2013). Small-molecule modulation of neurotrophin receptors: A strategy for the treatment of neurological disease. Nat. Rev. Drug Discov..

[54] Scarpi D., Cirelli D., Matrone C., Castronovo G., Rosini P., Occhiato E.G., Romano F., Bartali L., Clemente A.M., Bottegoni G. (2012). Low molecular weight, non-peptidic agonists of TrkA receptor with NGF-mimetic activity. Cell Death Dis..

[55] Finkbeiner S., Tavazoie S.F., Maloratsky A., Jacobs K.M., Harris K.M., Greenberg M.E. (1997). CREB: A major mediator of neuronal neurotrophin responses. Neuron.

[56] Bullitt E. (1990). Expression of C-fos-like protein as a marker for neuronal activity following noxious stimulation in the rat. J. Comp. Neurol..

[57] Shen M., Greenberg M. (1990). The Regulation and Function of c-fos and Other Immediate in the Nervous System. Cell.

[58] Curran T., Morgan J.I. (1985). Superinduction of c-fos by nerve growth factor in the presence of peripherally active benzodiazepines. Science (80-.).

[59] Milbrandt J. (2006). Nerve growth factor rapidly induces c-fos mRNA in PC12 rat pheochromocytoma cells. Proc. Natl. Acad. Sci. USA.

[60] Guo W., Ji Y., Wang S., Sun Y., Lu B. (2014). Neuronal activity alters BDNF–TrkB signaling kinetics and downstream functions. J. Cell Sci..

[61] Pongrac J.L., Rylett R.J. (2003). Molecular Mechanisms Regulating NGF-Mediated Enhancement of Cholinergic Neuronal Phenotype: c-Fos Trans-Activation of the Choline Acetyltransferase Gene. J. Mol. Neurosci..

[62] Blusztajn J.K., Berse B. (2000). The cholinergic neuronal phenotype in Alzheimer’s disease. Metab. Brain Dis..

[63] Perez-Lloret S., Barrantes F.J. (2016). Deficits in cholinergic neurotransmission and their clinical correlates in Parkinson’s disease. npj Park. Dis..

[64] Salehi A., Wesson Ashford J., Mufson J.E. (2015). Editorial (Thematic Issue: The Link between Alzheimer’s Disease and Down Syndrome. A Historical Perspective). Curr. Alzheimer Res..

[65] Zuccato C., Cattaneo E. (2009). Brain-derived neurotrophic factor in neurodegenerative diseases. Nat. Rev. Neurol..

[66] Michael G.J., Averill S., Nitkunan A., Rattray M., Bennett D.L., Yan Q., Priestley J. (1997). V Nerve growth factor treatment increases brain-derived neurotrophic factor selectively in TrkA-expressing dorsal root ganglion cells and in their central terminations within the spinal cord. J. Neurosci..

[67] María Frade J., Rodríguez-Tébar A., Barde Y.-A. (1996). Induction of cell death by endogenous nerve growth factor through its p75 receptor. Nature.

[68] Sofroniew M.V., Galletly N.P., Isacson O., Svendsen C.N. (1990). Survival of adult basal forebrain cholinergic neurons after loss of target neurons. Science.

[69] Latina V., Caioli S., Zona C., Ciotti M.T., Amadoro G., Calissano P. (2017). Impaired NGF/TrkA Signaling Causes Early AD-Linked Presynaptic Dysfunction in Cholinergic Primary Neurons. Front. Cell. Neurosci..

[70] Wu C.K., Yeh H.H. (2005). Nerve growth factor rapidly increases muscarinic tone in mouse medial septum/diagonal band of Broca. J. Neurosci..

[71] Heldin C.H. (1995). Dimerization of cell surface receptors in signal transduction. Cell.

[72] Xie Y., Tisi M.A., Yeo T.T., Longo F.M. (2000). Nerve Growth Factor (NGF) Loop 4 Dimeric Mimetics Activate ERK and AKT and Promote NGF-like Neurotrophic Effects. J. Biol. Chem..

[73] Brahimi F., Liu J., Malakhov A., Chowdhury S., Purisima E.O., Ivanisevic L., Caron A., Burgess K., Saragovi H.U. (2010). A monovalent agonist of TrkA tyrosine kinase receptors can be converted into a bivalent antagonist. Biochim. Biophys. Acta.

[74] Hanson L.R., Frey W.H. (2008). Intranasal delivery bypasses the blood-brain barrier to target therapeutic agents to the central nervous system and treat neurodegenerative disease. BMC Neurosci..

[75] Freiherr J., Hallschmid M., Frey W.H., Brünner Y.F., Chapman C.D., Hölscher C., Craft S., De Felice F.G., Benedict C. (2013). Intranasal insulin as a treatment for alzheimer’s disease: A review of basic research and clinical evidence. CNS Drugs.

[76] Tirassa P. (2011). The nerve growth factor administrated as eye drops activates mature and precursor cells in subventricular zone of adult rats. Arch. Ital. Biol..

[77] Lambiase A., Coassin M., Sposato V., Micera A., Sacchetti M., Bonini S., Aloe L. (2007). NGF topical application in patients with corneal ulcer does not generate circulating NGF antibodies. Pharmacol. Res..

